# Immunity Against Tetanus Infection, Risk Factors for Non-Protection, and Validation of a Rapid Immunotest Kit among Hospitalized Children in Nigeria

**DOI:** 10.3389/fneur.2013.00142

**Published:** 2013-09-24

**Authors:** Adebola Emmanuel Orimadegun, Bose Etaniamhe Orimadegun, Akinlolu Adedayo Adepoju

**Affiliations:** ^1^Institute of Child Health, College of Medicine, University of Ibadan, Ibadan, Nigeria; ^2^Department of Chemical Pathology, College of Medicine, University of Ibadan, Ibadan, Nigeria; ^3^Department of Paediatrics, College of Medicine, University of Ibadan, Ibadan, Nigeria

**Keywords:** protective immunity, tetanus, tetanos quick stick, hospitalized children, rapid screening test

## Abstract

Seroepidemiological studies of tetanus in Africans have focused mainly on adults especially pregnant women and data on children are scarcely reported. We investigated the seroprevalence of protective immunity level, determined risk factors for non-protection against tetanus, and evaluated the performance of Tetanos Quick Stick^®^ (TQS) among hospitalized children aged 1–9 years in Nigeria. Blood IgG antibody levels to tetanus was determined using enzyme-linked immunosorbent assay (ELISA) in the laboratory and TQS (an immunochromatographic test) at the bedside for 304 children admitted into emergency unit of a tertiary hospital in Ibadan, Nigeria. Demographic information and vaccination history were also collected. TQS results were compared with anti-tetanus antibody measured by ELISA using seroprotection cut-off of 0.1 IU/ml. Seroprevalence of protective level of immunity against tetanus using ELISA and TQS methods was 44.7 and 45.4% respectively. Protective level of immunity increased as age increases. Of the seven potential factors assessed, male gender and being second or more position among mother’s children were independent predictors of non-protective level of immunity. Absence of history of recent tetanus toxoid injection was significantly associated with non-protective level of immunity in univariate analysis but not logistic regression model. The agreement between the ELISA and the TQS results was good with a *k* coefficient of 0.931. TQS sensitivity was 95.7%, specificity 97.6%, positive predictive value 98.0%, and negative predictive values 96.0%. This study showed that lack of protective immunity against tetanus is common; few demographic characteristics correctly predict non-protection and IgG antibody levels to tetanus was accurately detected by TQS.

## Introduction

Tetanus is an acute neurological but vaccine-preventable disease caused by *Clostridium tetani* that causes significant morbidity and mortality among children in developing countries (1). A recent systematic analysis of data from 2000 to 2010 showed that neonatal tetanus decreased in Africa at an annual rate sufficient to attain the Millenium Development Goal 4 and it accounted for 20,000–276,000 neonatal deaths (1% of all child mortality) in 2010 (2). On the other hand, post-neonatal tetanus accounted for less than 1% of global child mortality (2). In Nigeria, of the five million babies born annually, 240,000 (4.8%) die within the first 4 weeks of life and tetanus accounts for up to 20% of these deaths (3).

Total eradication of tetanus is not likely because tetanus spores are found everywhere in the soil and stool of animals, but vaccination against the diseases can effectively protect susceptible individuals (4). The World Health Organisation (WHO) recommends administration of three doses of tetanus toxoid (TT) as one of the vaccines for routine immunization program to all infants and booster doses during adolescence and pregnancy in order to achieve elimination of tetanus in early childhood (5). Many of the countries in sub-Saharan African are implementing this recommendation and they have either attained the elimination of tetanus or making substantial progress toward achieving this goal but some are not (6). Nigeria is one of the 27 countries currently accounting for 90% of the global burden of the disease (6–9).

Despite the recent scaling up of routine immunization program in Nigeria, tetanus continues to affect children and many are dying. For instance, post-neonatal tetanus is a significant cause of emergency admission and a major killer of infants in Ibadan, Nigeria. An anecdotal observation of admissions into the children emergency ward of the University College Hospital (UCH) revealed that 16 out 767 children aged 1–9 years admitted in 2011 had tetanus (2.1%) and 9 of these cases died giving a case fatality rate of 56.3% (unpublished data). The persistence of tetanus and the high case fatality rate in spite of the availability of potent vaccine underscore the need to do seroepidemiological studies. Children presenting in the hospitals for whatever reason, especially the emergency unit provide a good opportunity to identify those who are not protected against tetanus and they could be offered the vaccine (10). A history of vaccination is often obtained by physician for the purpose of making decision but this does not necessarily imply that the patient had attained protective immunity (11). Instead of proper identification, many physicians habitually administer vaccines and tetanus immunoglobulin whether or not there is sufficient evidence to do so. Though the risk of injection of human blood products may be small but should nonetheless, not encourage its prescription. With good diagnostic tests to determine tetanus immunity status accurately, this practice and its potential risks could be averted.

The Enzyme-Linked Immunosorbent assay (ELISA) test can be used as the gold standard, with a cut-off value of 0.1 IU/ml for anti-tetanus IgG level, to define protection against tetanus (5). This cut-off is considered being protective in many studies and has been described as adequate (5, 11–14). However, ELISA test is not readily available in health centers in many developing countries where tetanus is still a public health problem; immunization status in this setting is normally assessed from the immunization history reported by the caregivers of children, which has been shown to be inaccurate (14, 15). The consequences of this are over- and under-prescription of vaccine and tetanus immunoglobulin, a human blood product. The determination of the level of serum anti-TT antibodies by an ELISA method allows an objective evaluation of tetanus immunity; however, this technique requires a blood sample and several hours of highly technical work, which cannot meet the emergency units’ requirements. An alternative to ELISA test is an immunochromatographic test [Tetanos Quick Stick^®^ (TQS), Gamma, Angleur, Belgium], which makes the semi-quantitative evaluation of anti-tetanus immunity possible at the bedside is now available. This test can be carried out on serum or whole blood, and can be performed on a drop of blood from a finger prick. TQS has been shown to be reliable when compared with ELISA in previous studies (12, 13, 16) but not in Nigeria. The present study was carried out to investigate the prevalence of protective level of immunity and risk factors for non-protection against tetanus infection among hospitalized children aged 1–9 in Nigeria. The performance of TQS in determining tetanus immunity was also evaluated against ELISA method.

## Materials and Methods

### Study design and patients recruitment

This prospective observational study was performed in the children emergency ward of the UCH, Ibadan, Nigeria. The UCH is located in an urban area of Ibadan city and had an average of 2500 yearly admissions. Children aged 1–9 years admitted during a 6-month period (April to September, 2012) were consecutively recruited into the study. To avoid interference with the current prophylaxis policy of the hospital, patients presenting with wounds were excluded from the study. Other exclusion criteria were persons under 1 year, prior inclusion in the same study and those who presented with tetanus. Of the 337 eligible patients, 304 participated in the study after their mothers or caregivers signed the informed consent form.

### Sample size justification

We postulated that the prevalence of protective immunity against tetanus among children to be 45.3% [based on a survey by Brabin et al. (17)]. We calculated that recruiting 304 out of 375 eligible gives an absolute precision of 2.43% and at 95% confidence level using the Win Episcope 2.0 software.

### Data collection

An interviewer-administered questionnaire was used to obtain demographic information and vaccination history. A child was considered to be fully immunized if he or she had received all of the following vaccines: a dose of Bacille Calmette Guerin (BCG), four doses of oral polio (OPV), three doses of diphtheria, pertussis, and tetanus (DPT), three doses of hepatitis B (HBV), and one dose of measles by the age of 12 months. Presentation of a vaccination card or any other official document concerning vaccination history was noted in the questionnaire. Sociodemographic characteristics (gender, age, number of siblings, position among mother’s children, ethnicity, and parents’ educational level and occupation) and history TT injection 1 year before the study were also collected. Caregivers were questioned about past medical history (medical and surgical antecedents), date of last trauma with or without a wound, date of childbirth, and consultation with a general practitioner to corroborate claims of tetanus vaccinations. A 3 ml blood sample was collected from each participant into a plain specimen bottle and serum was obtained and kept in kept at −20°C until laboratory analysis. Weight and height of all participants were measured and compared with WHO reference population to obtain the *z*-scores for weight-for-height using the WHO AnthroPlus, a software for the global application of the WHO Reference 2007 for monitoring the growth of school-age children and adolescents as described previously (18).

### Evaluation of tetanus immunity

Immunity against tetanus was evaluated using ELISA in the laboratory and TQS by the bedside. Anti-tetanus IgG concentrations were measured by ELISA as described in two previous studies (15, 19). Concisely, sera were incubated on TT-coated ELISA plates. Fixed IgG was revealed by the addition of peroxidase-conjugated anti-human IgG after incubation and washing. The anti-tetanus IgG concentrations of the samples were assessed by reference to a standard curve constructed from serial dilutions of a preparation of human tetanus immunoglobulin with a known concentration of anti-tetanus IgG (Tetabulin 250, Baxter Bioscience, and Deerfield, IL, USA).

The TQS is based on an immunochromatographic method which has been described in a previous study (11). All TQS tests were performed using whole blood, and were interpreted after 10 min according to the manufacturer’s recommendation. Results (whether positive, negative, or equivocal) were recorded in the patient’s questionnaire. A research assistant was trained on the use and interpretation of the TQS results before starting the study. All tests kits were carefully preserved and reviewed by the investigators to ascertain correctness of the results. Six tests reported as equivocal by the assistant nurse were repeated by the main investigator (AEO) who decided they were positive result without being aware of the ELISA result.

### Ethical considerations

Ethical approval was obtained from the Oyo State Research Ethical Review Committee, Ministry of Health, Oyo state secretariat, Ibadan. Written informed consent was obtained from the participants’ parents or caregivers after the purpose of the study was explained to each of them in the language they understood. Serial numbers and codes were used to identify participants. The questionnaires were kept in a secured place accessible to the researcher alone.

### Data analysis

Socioeconomic classes of participants were assessed using the method employed by Oyedeji (20) in a similar setting. Data were first analyzed using descriptive statistics [medians, interquartile range (IQR), frequencies]. Seroprevalence among the different categories were calculated. Bivariate analysis was used to assess associations between various potential risk factors and protection based on ELISA results. Those factors with significant associations were included in a logistic regression analysis to exclude the effects between the variables and to identify independent predictors of a non-protective antibody level. *p*-Values less than 0.05 were considered significant. These statistical analyses were performed with SPSS 17.0 for Windows (SPSS, Chicago, IL, USA). The performance of immunity status determined using TQS was assessed by determining the sensitivity, specificity, positive and negative predictive values (NPV), with 95% confidence intervals (CI). ELISA was considered to be the gold standard. We used a threshold of 0.1 IU/ml of serum to define positive results in ELISA.

## Results

### Characteristics of study population

**Figure 1:**
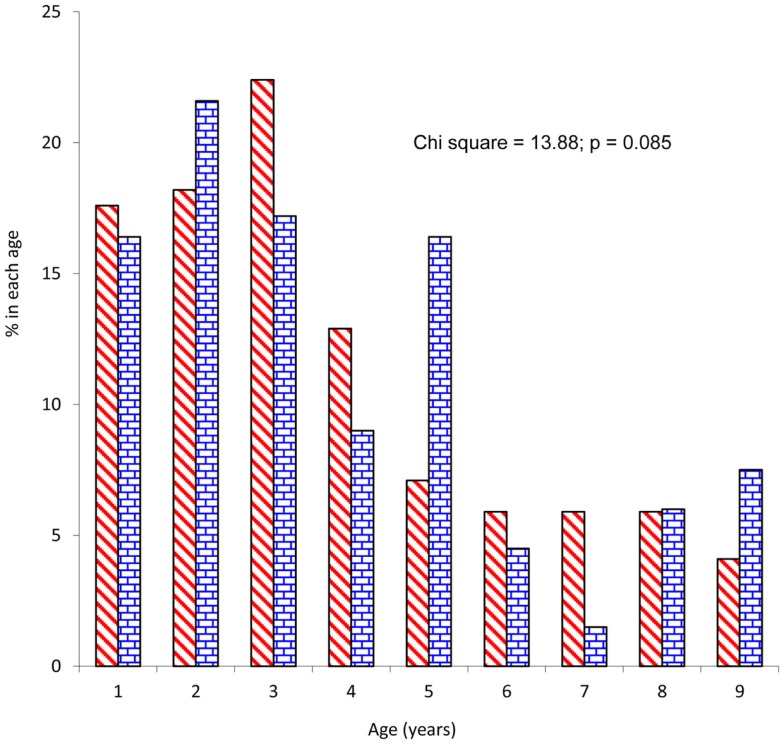
**Distribution of study patients by age**.

The study patients comprised 170 (55.9%) males and 134 (44.1%) females giving a male to female ratio of 1.3:1. Mean age of participants was 3.7 ± 2.3 years and children 5 years and below constituted 79.3%. There was no significant difference between male and female patients’ distribution by age (Figure 1). Mean age (3.6 ± 1.1 years) and gender distribution (40 male and 31 female) of children who did not participate in the study were not statistically different from those of the study patients. Of the 304 patients, 24 (7.9%), 98 (32.2%), and 182 (59.9%) were from high, middle, and low socioeconomic classes and majority (80.3%) were from the Yoruba ethnic group. Forty-six percent (*n* = 140) of the study patients were first child of their mothers and median numbers of children in the families was 3 (range = 1–7). Almost a quarter (24.7%) of the study patients were underweight (weight-for-height *z*-score <2.0).

### Tetanus immunization history of study participants

Table 1 displays the tetanus immunization history of the study patients. History of vaccination was confirmed by examining the vaccination card and history taking in 196 (64.5%) and obtained from only history taking from the caregivers in 108 (35.5%) of patients who participated in the study. Only 90 (29.6%) of study patients were fully vaccinated according to the Nigeria National Programme on Immunization (NPI) by the age of 10 months. Uptake rate for DPT-3 was 19.1% as indicated by vaccination card and 29.9% as indicated by history. Combining evidence from vaccination card and history suggested a total DPT-3 uptake rate of 49.0%. Only 25 (8.2%) participants had received at least a booster dose of TT injection after routine DPT vaccinations in infancy. Stratifications of tetanus-related vaccination by gender and birth position were as shown in Table 2. There is no association between vaccination and gender as well as birth position.

**Table 1:** **Tetanus-related vaccination status of study patients**.

	Vaccination card seen (*n* = 196)			Obtained history only (*n* = 108)			Total (*n* = 304)	
	*n*	% of 196	% of 304	*n*	% of 108	% of 304	*n*	%
Completed immunization	28	14.3	9.2	62	57.4	20.4	90	29.6
**TETANUS ANTIGEN**								
DPT-1	164	83.7	53.9	105	97.2	34.5	269	88.5
DPT-2	94	48.0	30.9	99	91.7	32.6	193	63.5
DPT-3	58	29.6	19.1	91	84.3	29.9	149	49.0
Recent TT injection	8	4.1	2.6	17	15.5	5.6	25	8.2

**Table 2:** **Tetanus-related vaccination status by gender and birth position of study patients**.

Tetanus antigen	Gender					Birth position				
	Male (170)		Female (134)		*p*	First born (140)		Not first born (164)		*p*
	*n*	%	*n*	%		*n*	%	*n*	%	
DPT-1	148	87.1	121	90.3	0.189	124	88.6	145	88.4	0.483
DPT-2	110	64.7	83	61.9	0.309	91	65.0	102	62.2	0.306
DPT-3	81	47.6	68	50.7	0.296	69	49.3	80	48.8	0.465

### Prevalence of anti-tetanus protection among study participants

Overall, the prevalence of seroprotection by ELISA was 44.7% (95% CI = 39.1, 50.5%) and the prevalence of seroprotection by TQS tests was 45.4% (95% CI = 39.7, 51.2%). Using ELISA test results, proportions of children with seroprotection were significantly more in females (74/134) than males (62/170), *p* = 0.001; children who had history of recent TT injection (17/25) than those who did not (119/279), *p* = 0.015; and among first born of mothers (78/140) than others (58/164), *p* < 0.001. However, family socioeconomic class, ethnicity, and nutritional status (defined using weight-for-age) were not significantly associated with seroprotection against tetanus. Age-specific prevalence and risk of non-protective tetanus immunity by age as detected by ELISA and TQS were as shown in Table 3. For both ELISA and TQS test results, a statistically significant trend was demonstrated with prevalence of non-protection increasing as age increases. Participants aged 8 years had the highest prevalence of non-protection against tetanus.

**Table 3:** **Prevalence and risk of non-protective tetanus immunity by age of study patients**.

Age (years)	*N*	Non-protection detected by TQS		Non-protection detected by ELISA	
		*n* (%)	OR (95% CI)	*n* (%)	OR (95% CI)
1	52	22 (42.3)	1.00	24 (46.2)	1.00
2	60	24 (40.0)	0.91 (0.43, 1.93)	24 (40.0)	0.78 (0.37, 1.65)
3	61	33 (54.1)	1.61 (0.76, 3.39)	35 (57.4)	1.57 (0.75, 3.31)
4	34	26 (76.5)	4.43 (1.69, 11.63)	25 (73.5)	3.24 (1.27, 8.27)
5	34	24 (70.6)	3.27 (1.30, 8.21)	24 (70.6)	2.80 (1.12, 7.01)
6	16	8 (50.0)	1.36 (0.44, 4.20)	8 (50.0)	1.17 (0.38, 3.58)
7	12	6 (50.0)	1.36 (0.39, 4.80)	6 (50.0)	1.17 (0.33, 4.10)
8	18	16 (88.9)	10.91 (2.27, 52.41)	15 (83.3)	5.83 (1.51, 22.60)
9	17	7 (41.2)	0.95 (0.31, 2.90)	7 (41.2)	0.82 (0.27, 2.48)
Total	304	166 (54.6)	–	168 (55.3)	–

Chi square for trend = 6.672; *p* = 0.009 for TQS.

Chi square for trend = 4.081; *p* = 0.043 for ELISA.

### TQS validation against the ELISA

**Figure 2:**
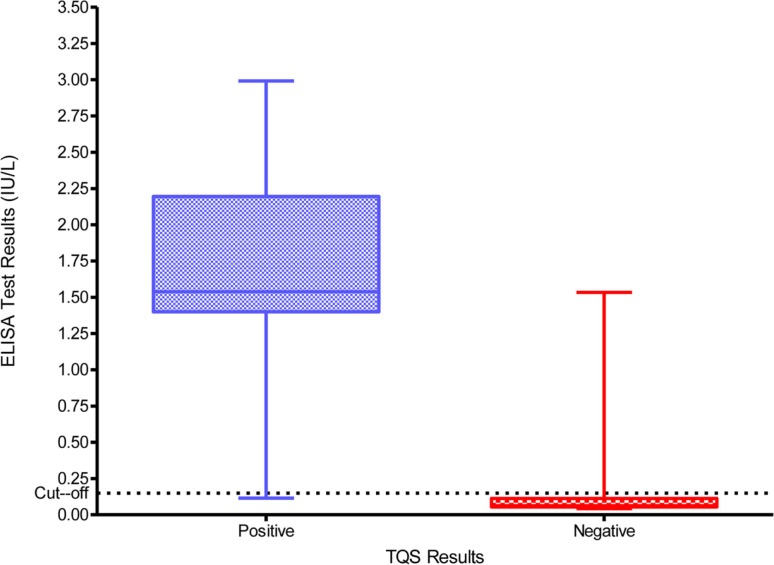
**Distribution of the anti-tetanus toxoid IgG titers as determined by ELISA among TQS positive and negative patients**.

The ELISA anti-tetanus IgG levels ranged from 0.0064 to 2.4441 IU/ml and the distribution of these levels according to the initial interpretation of the TQS (positive and negative) is summarized in Figure 2. The box plot (Figure 2) represents the 25th and 75th percentiles, the line inside the box represents the median and the lower and upper bars correspond to the 2.5th and 97.5th percentiles, respectively. The dotted line at 0.1 IU/ml represents the cut-off that defines tetanus seroprotection. The median anti-tetanus IgG level for patients with a positive TQS of 1.5380 IU/ml (IQR = 1.2677–1.7353) was considerably higher than for those with a negative TQS of 0.0803 IU/ml (IQR = 0.0464–0.6911). Table 4 shows the comparison between TQS and the anti-tetanus IgG concentrations measured by ELISA method using the cut-off of 0.1 IU/ml. The agreement between the ELISA and the TQS results was good with a *k* coefficient of 0.93 (*p* < 0.001). On the basis of Table 4, the TQS sensitivity = 97.1% (95% CI = 94.2, 99.9%), specificity = 96.4% (95% CI = 93.6, 99.2%), positive predictive value (PPV) = 95.7.0% (95% CI = 92.3, 99.1%), and NPV = 97.6% (95% CI = 95.3, 99.9%).

**Table 4:** **Comparison between TQS and the anti-tetanus toxoid IgG concentrations measured by ELISA method (threshold 0.11 IU/ml)**.

	Anti-tetanus toxoid IgG concentrations		Total
	≥0.11 IU/ml	<0.11 IU/ml	
TQS positive	132 (95.7%)	6 (4.3%)	138 (100.0)
TQS negative	4 (2.4%)	162 (97.6%)	166 (100.0)
Total	136 (44.7%)	168 (55.3%)	304 (100.0)

### Identification of predictive factors for non-protection against tetanus

Out of seven factors investigated in this study, gender, age, position of the participants among mother’s children, and history of recent TT injection were significantly associated with seroprotection in bivariate analysis (Table 5). Among the four variables stated above, only three were identified as independent predictors of non-protective antibody level (Table 5). Sex had the strongest influence on level of immunity against tetanus. A male compared to a female child was 2.37 times more and not being the first compared to being the first child was 2.10 times more likely to have non-protective immunity against tetanus. Also, an increase of 1-year in age has a 15% (95% CI 4–28%) increase in odds of having non-protective immunity against tetanus.

**Table 5:** **Potential risk factors for non-protective tetanus immunity among. study patients**.

Factors	*N*	Not protected		UOR		AOR	
		*n*	%	OR	95% CI	OR	95% CI
**GENDER**							
Male	170	108	63.5	2.15	1.35, 3.41	2.37	1.45, 3.87
Female^a^	134	60	44.8	1	–	1	–
Age (years)^b^	304	168	55.3	1.11	1.01, 1.22	1.15	1.04, 1.28
**POSITION**^c^							
First born^a^	140	62	44.3	1	–	1	–
Not first	164	106	64.6	2.29	1.45, 3.65	2.10	1.26, 3.51
**HISTORY RECENT TT**							
Yes^a^	25	8	32.0	1		1	–
No	279	160	57.3	2.86	1.19, 6.84	0.38	0.15, 1.00
Ethnicity
Yoruba	244	138	56.6	1.30	0.74, 2.29		
Non-Yoruba^a^	60	30	50.0	1	–		
**PARENTS’ SOCIAL CLASS**							
High^a^	24	12	50.0	1	–		
Middle	98	55	56.1	1.28	0.53, 3.13		
Low	182	101	55.5	1.25	0.53, 2.92		
**NUTRITIONAL STATUS**							
WAZ < 2.0	75	46	61.3	1.39	0.82, 2.37		
WAZ ≥ 2.0^a^	229	122	53.3	1	–		

^a^Reference category

^b^Continuous variable

^c^Position among mother’s children.

UOR, unadjusted odds ratio; AOR, adjusted odds ratio; WAZ, weight-for-age *z*-score.

## Discussion

This study has revealed that the prevalence of protection against tetanus among children hospitalized in a tertiary hospital in Nigeria using ELISA and TQS methods was 44.7 and 45.4% respectively; and TQS is good test kit for assessing tetanus immunity. Also, the data showed that gender, age, and position of the child (whether first or not) independently predicted non-protective level of immunity against tetanus. The lack of association between tetanus-related immunization status and gender nor birth position suggests that the significantly higher seroprotection in females and in first born children could not have been because they were more likely to be vaccinated. The low proportion of children with protective level of immunity against tetanus in this study underscores the need to reappraise the current childhood tetanus immunization policy in Nigeria. Whilst the DPT-3 coverage rate among these hospitalized children was 49.0%, we found out that 64.5% of the children had DPT-3 as evidenced by immunization card. This immunization rate is considerably less than the recommended 80% required for the entire country (21), but it is higher than full immunization rate of 23% currently reported for Nigeria (22). High DPT-1 to DPT-3 vaccine uptake dropout rate found in the study had also been previously reported (23). Oladokun et al. (23) reported a dropout rate of 32.1% among children of female traders in the same city of Ibadan, Nigeria.

That risk of non-protection against tetanus increases with increasing age was not surprising. Previous reports have shown that immunity against tetanus wanes with increasing age (5). Many of the caregivers who participated in the study could not provide tetanus immunization card, therefore it may be rational to administer booster dose of TT to children in similar situation, especially if they are male, not first born of their mothers and history of recent TT injection is lacking. In addition, children who present with tetanus-prone wound in the emergency unit would benefit from anti-tetanus immunoglobulin (5). The strategy of immunizing at every opportunity is recommended by the Global Advisory Group of the WHO Expanded Programme on Immunization (EPI) since 1983 (24). However, this is not currently been done for tetanus vaccination in children in many health facilities. Given the low level of seroprotection among the study patients, immunizations need to be offered at every contact point, including preventive and curative health services. An opportunity for immunization is considered missed when a person who is eligible for immunization and who has no contraindication to immunization visits a health service and does not receive all the needed vaccines. In Nigerian settings, eliminating missed opportunities may likely raise the overall immunization coverage.

There is no universal agreement regarding the absolute seroprotective level of immunity against tetanus. Protection is only observed when there are enough antibodies to neutralize the toxin with regard to the amount of toxin (5). The choice of the cut-off for protective level of anti-tetanus used in this study was based on the best compromise between the sensitivity and the specificity as defined by receiver–operator characteristic curves, as well as by the work of Simonsen et al. (15). These authors (15) also demonstrated that the correlation between the ELISA method and *in vivo* seroneutralization techniques was poor above this threshold for individuals with incomplete vaccination. Since appropriate prophylaxis is the basis of tetanus prevention based on the characteristics of the wound and on the patient’s immunity status, it is important to device an objective method of deciding who require tetanus immunoglobulin injection and/or toxoid. Currently in Nigeria, evaluation of immunity only takes the vaccination history into account. Thus, the TQS test could be helpful in assessing immunity against tetanus by age in an effort to fine-tune the optimal timing of booster doses in Nigeria.

This present study shows that TQS could be a good tool in the evaluation of immunity against tetanus in children. The test was validated using the ELISA method as the gold standard. The agreement between the two tests was high, given the *k* concordance coefficient of 0.93. This value is higher than 0.71 (14), and 0.77 (16) found in a similar evaluations. The sensitivity (95.7%) was higher than that found in other studies (12–14). The NPVs 96.0% and a negative TQS test would be false negative in only 2.4% of cases with the practical consequence of giving an unnecessary anti-tetanus prophylaxis. However, an over-protected child is evidently more tolerable than a patient wrongly considered to be protected. Moreover, a specificity of 97.6% and a PPV of 98.0% implying that a positive test is thus a true positive in 95.7% of cases in the observed seroprevalence, when a threshold of 0.1 IU/ml is chosen. These values are comparable to reported values in other studies using the same threshold (12–14). The implications of these performance results are: a patient with a positive test had a 98.0% probability of having an antitoxin level higher than 0.1 IU/ml while a negative test indicated a 96.0% probability of having an anti-tetanus level less than 0.1 IU/ml. TQS thus provides an objective evidence for assessment of tetanus immunity. In situations of incorrect or doubtful history from caregivers, its use could improve the administration of anti-tetanus prophylaxis.

The high performance of the TQS test by the bedside obtained in this study confirms the importance and benefit of this test over the usual clinical history taking. However, its use in children emergency units should not be considered without a quality assurance process and corroboration of both laboratory and clinical judgments. There is the need to conduct a cost-effectiveness study to compare the different strategies for tetanus prophylaxis treatment for wounds; test all, or treat those who are suspected to be unprotected. Compared to ELISA technique, used to measure the level of antibody against tetanus, TQS offers some advantages: the result is quick, relatively painless, and does not require venous blood sampling. The sampling of a drop of capillary blood at the fingertip is a relatively less invasive procedure and may be more acceptable to children than venepuncture. The TQS test is practicable at the bedside by any healthcare provider in an emergency unit. Nevertheless, it will cost about eight US Dollars (approximately 1,500 Naira) but this is not in any way comparable to high cost of treating tetanus and high mortality rate. Another challenge with TQS is that interpretation of the test is not always easy. Equivocal TQS results are not infrequent (13, 14). Indeed, six tests were interpreted as equivocal (2%) by the research assistant in this study. In our experience, a discontinuous or whitish strip which is difficult to visualize occurred occasionally with intermediate/declining protection. In such cases, it is safer to consider TQS as negative and boost the child’s immunity, especially in wounds that are difficult to clean. With good training, TQS is reproducible by all healthcare professionals (25).

One factor that could limit the interpretation and generalization of data presented in this study is that 33 (10.9%) of eligible children did not participate. Though age and sex distributions of these children were similar to participants’, it was impossible to assess their level immunity against tetanus. It therefore remains unknown from the data whether their inclusion in the study might have altered the results, especially the prevalence estimates. There is the need for further study to estimate the number of cases in which the TQS result would have modified the decision to give prophylaxis on the basis of the history in patients and cost-benefit analysis. Another factor that needs to be considered in the interpretation of data on seroprotection in this study is the predominance of children 5 years and below in the study population. However, the pattern of age distribution observed in this study is similar to what has been previous reported in the same emergency unit for many other disease conditions (26, 27).

In conclusion, lack of protective immunity against tetanus is frequent among hospitalized children in Nigeria. Age, gender, and position among mother’s children were identified as independent predictive factors for non-protective level of immunity. In this study, because the WHO schedule for infant DTP doses recommends an accelerated schedule with doses given prior to full immune system maturity (4), there are good grounds to be concerned that immunity wanes relatively early in childhood. The TQS proved to be a reliable tool for the evaluation of immunity to tetanus. The use of TQS, in combination with the vaccination history has the potential to improve evaluation of tetanus immunity, which is essential to optimize prophylaxis policy in Nigerian children. However, bearing in mind the possibility of false positive results with TQS, we suggest anti-tetanus immunoglobulin or toxoid be given to children who cannot afford the cost of testing for immunity level and has any of the identified risk factors for non-protection.

## Conflict of Interest Statement

The authors hereby declare no competing interests. The manufacturer of TQS had no involvement in the study design; in the collection, analysis, and interpretation of data; in the writing of the report; and in the decision to submit the paper for publication.
